# Efficacy and Cost-Effectiveness Analysis of Internet-Based Cognitive Behavioral Therapy for Obsessive-Compulsive Disorder: Randomized Controlled Trial

**DOI:** 10.2196/41283

**Published:** 2023-05-24

**Authors:** Yiwen Wu, Xin Li, Yuxin Zhou, Rui Gao, Kaifeng Wang, Huiling Ye, Na Lyu, Chun Wang, Ning Zhang, Zhen Wang, Qing Fan

**Affiliations:** 1 Shanghai Mental Health Center Shanghai Jiao Tong University School of Medicine Shanghai China; 2 Xinhua Hospital Shanghai Jiao Tong University School of Medicine Shanghai China; 3 Department of Psychiatry Brain Hospital Affiliated to Nanjing Medical University Nanjing China; 4 Shanghai Intelligent Psychological Evaluation and Intervention Engineering Technology Research Center Shanghai China; 5 Shanghai Key Laboratory of Psychotic Disorders Shanghai China; 6 Mental Health Branch China Hospital Development Institute Shanghai Jiao Tong University Shanghai China

**Keywords:** obsessive-compulsive disorder, cognitive behavioral therapy, internet-based cognitive behavioral therapy, cost-effectiveness, randomized controlled trial

## Abstract

**Background:**

Obsessive-compulsive disorder (OCD) is a common and chronic mental illness with a high rate of disability. Internet-based cognitive behavioral therapy (ICBT) makes online treatment available to patients and has been shown to be effective. However, 3-arm trials on ICBT, face-to-face cognitive behavioral group therapy (CBGT), and only medication are still lacking.

**Objective:**

This study is a randomized, controlled, assessor-blinded trial of 3 groups for OCD: ICBT combined with medication, CBGT combined with medication, and conventional medical treatment (ie, treatment as usual [TAU]). The study aims to investigate the efficacy and cost-effectiveness of ICBT related to CBGT and TAU for adults with OCD in China.

**Methods:**

In total, 99 patients with OCD were selected and randomly assigned to the ICBT, CBGT, and TAU groups for treatment for 6 weeks. The primary outcomes were the Yale-Brown Obsessive-Compulsive Scale (YBOCS) and the self-rating Florida Obsessive-Compulsive Inventory (FOCI), compared at baseline, during treatment (3 weeks), and after treatment (6 weeks), to analyze efficacy. The secondary outcome was the EuroQol Visual Analogue Scale (EQ-VAS) scores of the EuroQol 5D Questionnaire (EQ-5D). The cost questionnaires were recorded to analyze cost-effectiveness.

**Results:**

Repeated-measures ANOVA was used for data analysis, and the final effective sample size was 93 (ICBT: n=32, 34.4%; CBGT: n=28, 30.1%; TAU: n=33, 35.5%). After 6-week treatment, the YBOCS scores of the 3 groups significantly decreased (*P*<.001), and there were no significant differences among groups. The FOCI score of the ICBT (P=.001) and CBGT (P=.035) groups was significantly lower than that of the TAU group after treatment. The total cost of the CBGT group (renminbi [RMB] 6678.45, 95% CI 4460.88-8896.01 [US $1010.36, 95% CI 678.87-1345.84]) was significantly higher than that of the ICBT group (RMB 3308.81, 95% CI 2476.89-4140.73[US $500.58, 95% CI 374.72-626.43], *P*<.001) and the TAU group (RMB 2259.61, 95% CI 2074.16-2445.05 [US $341.85, 95% CI 313.79-369.90], *P*<.001) after treatment. The ICBT group spent RMB 303.19 (US $45.97) less than the CBGT group and RMB 11.57 (US $1.75) less than the TAU group for each unit reduction in the YBOCS score.

**Conclusions:**

Therapist-guided ICBT combined with medication is as effective as face-to-face CBGT combined with medication for OCD. ICBT combined with medication is more cost-effective than CBGT combined with medication and conventional medical treatment. It is expected to become an efficacious and economic alternative for adults with OCD when face-to-face CBGT is not available.

**Trial Registration:**

Chinese Clinical Trial Registry ChiCTR1900023840; https://www.chictr.org.cn/showproj.html?proj=39294

## Introduction

Obsessive-compulsive disorder (OCD) is a common and chronic mental illness with a high rate of disability [1]. The symptoms of OCD are mainly obsessions, compulsions, or both, and its remission rate is low [2], which greatly affects the work and social interaction of patients [3]. Finding effective interventions to treat OCD is important. According to National Institute for Health and Care Excellence (NICE) CG31 guidelines [4], cognitive behavioral therapy (CBT)/exposure and response prevention (ERP) and selective serotonin reuptake inhibitors (SSRIs) are currently the first-line strategies for the treatment of OCD in adults. However, conventional CBT still has limitations, such as a high dropout rate [5], high price, inconvenience [6], and stigma [7]. In addition, a lot of resources are consumed by patients with OCD to implement CBT. Research focusing on new methods of OCD treatment is needed.

Internet-based cognitive behavioral therapy (ICBT) makes online treatment available to patients. ICBT is divided into therapist-guided ICBT and self-guided ICBT. ICBT has the advantages of convenience and time saving, which can solve the problem of uneven distribution of medical resources and make up for the limitations of conventional CBT. The results of meta-analyses have shown that therapist-guided ICBT is as effective as face-to-face CBT (ffCBT) for various mental health conditions [8], including OCD [9]. The effectiveness of ICBT in the treatment of depression [10-12], anxiety [13], pediatric anxiety [14], and OCD [15] has been proven. In addition, ICBT has been proven to be a cost-effective treatment in earlier studies of mental illness [16,17].

ICBT for OCD has been generally proven to be effective. A randomized controlled trial (RCT) in 2013 showed that ICBT and the iCBT program in book format (bCBT) were more effective than the waiting treatment group (waitlist) in improving the obsessive-compulsive and depressive symptoms of patients with OCD [18]. An RCT in 2014 also found that the effectiveness of therapist-guided ICBT in improving obsessive-compulsive and depressive symptoms is significantly higher than that of conventional medical treatment (ie, treatment as usual [TAU]) [19]. The acceptance, feasibility, and effectiveness of therapist-guided ICBT were also verified in New York in 2017 [20]. The long-term maintenance of the efficacy of therapist-guided ICBT has also been proven [21,22]. In terms of health economics research on ICBT for OCD, Andersson et al. [23] collected health economics indicators through RCT research and found that ICBT is more cost-effective than online support therapy without CBT techniques. In comparison with waitlist controls, therapist-guided ICBT was more cost-effective due to the substantial societal cost savings generated by ICBT [24]. Compared with internet-based progressive relaxation therapy (iPRT) and ffCBT, therapist-guided ICBT was also the most cost-effective treatment method for OCD [25].

At present, although the number of international studies on the treatment of OCD with ICBT is gradually increasing, most RCTs conducted only set a waitlist or traditional medicine group to compare with ICBT and do not use ffCBT as the control group. In most cases, these waitlist controls are not restricted to take part in any psychotherapy or physiotherapy other than ffCBT, which introduces irrelevant variables. In addition, many current studies on ICBT for OCD are affected by comorbidities and heterogeneous medications. The latest ICBT research on OCD mainly focuses on children and adolescents, and there is a lack of research on adults. Up to now, there is no research on ICBT for OCD and its health economics analysis of OCD in China. Given these deficiencies, the efficacy and costs of ICBT for OCD in China deserve further research. Considering feasibility and cost-effectiveness, cognitive behavioral group therapy (CBGT) was set as the ffCBT active control group in this study. In a meta-analysis of CBT for OCD, individual CBT showed small and nonsignificant effect sizes (0.17) compared to CBGT and showed a noninferior efficacy of CBGT to individual CBT [5].The preexperiment of the study preliminarily demonstrated the efficacy of ICBT as being equal to that CBGT in the treatment of patients with OCD (men=20, women=8) for a period of 6 weeks [26]. This paper further explored the results after increasing the sample size and control group.

The study conducted an RCT to compare the feasibility, safety, efficacy, and cost-effectiveness of ICBT, CBGT, and TAU in patients with OCD. TAU involved the administration of psychotropic medications as usual. Both ICBT and CBGT were combined with medical treatment, and patients in the ICBT or the CBGT group also took psychotropic medications as usual. The study used therapist-guided ICBT because a meta-analysis showed that therapist-guided ICBT has better effects than self-guided ICBT [27].

The study aimed to investigate the efficacy and cost-effectiveness of ICBT related to CBGT and TAU for adults with OCD in China. The study was a noninferiority trial and hypothesized that the efficacy of therapist-guided ICBT is no less than that of face-to-face CBGT and medical treatment for OCD. In addition, ICBT was expected to be more cost-effective due to lower costs of treatment.

## Methods

### Trial Design

The study was a 6-week, assessor-blinded, clinical RCT with patients with OCD allocated 1:1:1 to the ICBT, CBGT, and TAU groups. Primary and secondary outcomes were measured 3 times: at baseline (0 weeks), during treatment (3 weeks), and after treatment (6 weeks). The study was carried out at the Shanghai Mental Health Center in Shanghai, China, and was registered in the Clinical Trial Registry (registration no. ChiCTR1900023840). Recruitment and intervention for the trial started in 2018.

### Ethical Considerations

The study adhered to the Consolidated Standards of Reporting Trials (CONSORT) and was approved by the Shanghai Ethical Review Committee (2018-57).

### Participants

The study subjects were 99 patients with OCD who were consecutively enrolled from a public psychiatric hospital. Information about the trial was sent to clinicians and advertised on posters in the clinic lobby and the online WeChat public account. Participation was referred by psychiatrists or via self-referral, and both were diagnosed as OCD by psychiatrists at the Shanghai Mental Health Center, China. After that, participants were asked to complete an online screening and a brief phone interview. Suitable participants were invited to take part in a face-to-face psychiatric assessment conducted by trained evaluators on duty to determine inclusion or exclusion. The evaluator used the Mini-International Neuropsychiatric Interview (MINI) [28] to rule out comorbidities and the Yale-Brown Obsessive-Compulsive Scale (YBOCS) [29] to assess obsessive-compulsive symptoms.

The inclusion criteria were(1) age between 18 and 54 years, (2) being satisfied with the diagnostic criteria for OCD in the *Diagnostic and Statistical Manual of Mental Disorders, Fifth Edition* (DSM-V) and taking medication stably, (3) YBOCS score≥16 and ≤31, and (4) education level≥6 years. The exclusion criteria were (1) severe comorbidity (ie, bipolar disorder, psychosis, alcohol and drug abuse, or acute suicidal ideation), (2) too severe obsessive-compulsive symptoms to be able to participate in the experiment, (3) high risk of suicide, (4) severe central system or physical disease, (5) pregnant women or women getting ready for pregnancy and lactating women, and (6) other treatments being performed.

### Interventions

All patients in the 3 groups had taken medication before the start of the experiment, 84.9% (n=79) of the patients had taken medication stably for more than 8 weeks, most of the remaining patients had taken medication stably for less than 8 weeks, and a few patients had discontinued medication before enrollment. The types of medications were SSRIs, and the types/doses were not changed during the study.

#### ICBT Intervention

Patients in the ICBT group used the Cognitive Behavioral Therapy China (CBTC) platform linked to the CBTC website [30] for modular treatment and completed 12 ICBTs under the guidance of a therapist. The platform could set up a training module according to the personalized situation of patients and convert the CBT module into an online mode. In the study, 5 intervention modules were set up, as shown in Table 1, and a total of 12 training sessions were conducted in 6 weeks.

**Table 1:** CBTC^a^ platform intervention modules.

Module	Training program	Homework	Number of training sessions (N=12), n (%)
1. Understanding OCD^b^ and subjective discomfort units	Psychological education of OCD	To understand the Subjective Units of Distress Scale (SUDS), symptom monitoring	2 (16.7)
2. Training methods and exposure checklist	Psychological education on ERP^c^	To create an exposure registration form	2 (16.7)
3. Reward list and exposure design	Introduction to exposure design	To create a reward list, establish a code of practice for ritual prevention, design for exposure	2 (16.7)
4. Exposure practice	Exposure content adjustment, use of exposure sheets for instruction	Exposure practice	5 (41.7)
5. Relapse prevention	Psychological education	To rebuild new rules	1 (8.3)

^a^CBTC: Cognitive Behavioral Therapy China.

^b^OCD: obsessive-compulsive disorder.

^c^ERP: exposure and response prevention.

The therapist conducted a pretreatment interview with each patient before and trained them all on how to use the CBTC platform for practice. A senior domestic OCD and ICBT therapist supervised the treatment. The patients used their accounts and passwords to log on to the platform and receive treatment for the module of facing OCD. There were in total 12 treatments, which lasted 6 weeks, about 120 minutes per treatment, twice a week. After each module treatment was completed, the platform arranged corresponding tasks. Before the next treatment, the patients filled in the completion status of the homework so that the therapist could obtain the severity of their symptoms in real time. The therapist provided feedback to the patients through the platform during the entire treatment process and answered questions on time, but the therapist allocated no more than 15 minutes to each patient per week.

Patients participating in ICBT needed to log on to the platform at least twice a week to complete their studies and the homework after each module. If a patient failed to log on to the platform for study or to complete the homework on time, the online therapist would send a short message to remind the patient. If the patient failed to log on to the platform or failed to complete the homework 3 times, the platform would automatically cancel the account and the patient would not be able to log on to the platform for treatment. For patients who missed a certain treatment for some reason or faced difficulties, the researcher would remind them to complete this treatment in time via phone and email.

#### CBGT Intervention

The CBGT group was headed by a nationally registered therapist who had received systematic training. The group was closed and homogeneous, and all face-to-face sessions were audio-recorded to ensure that therapists adhered to the treatment manual. Each subgroup included 6-8 patients, who were treated twice a week for a total of 12 treatments, each 120 minutes long, which lasted 6 weeks. A professional group cognitive therapist supervised this group during treatment. The treatment location was the group psychotherapy room of the Shanghai Mental Health Center. The treatment room was not changed during the treatment period.

The structure, content, and number of treatments followed the *Group Cognitive Behavioral Therapy of OCD – A Treatment Manual* [31], and the frequency of twice-weekly treatment was feasible and effective in our preliminary trials [26]. Before treatment, 2 interviews of 45 minutes each were conducted for assessment and psychoeducation. The first treatment was group establishment and psychological education. The second treatment was to develop an ERP plan. The third treatment started with the first ERP exercise. ERP exercises continued for the 4th-11th treatments. Feedback and a summary were provided at the end of each treatment, and the homework was reviewed in the next treatment. The 12th treatment included summaries and recurrence prevention. Although the group members were encouraged to discuss their practice difficulties and help each other in the 120-minute treatment time, which may be a special benefit of the CBGT group [32], the CBGT group had the same treatment times (12 sessions), treatment duration (6 weeks), treatment frequency (twice a week) and treatment modules (5 modules) as the ICBT group.

For handling other special situations, patients who were absent from a certain treatment for some reason were informed of the homework via telephone and email, and those who had difficulty completing the homework could listen to the recording with the researcher for the retrospective study. Patients were deemed to have dropped out of the study if they were absent 3 times in total.

#### TAU Intervention

The TAU group was treated with only medication. The medication interventions of the 3 groups were under the charge of an associate chief psychiatrist, who was unaware of the grouping of patients. The medicines used for the treatment of OCD in this study were SSRIs approved by the State Food and Drug Administration (SFDA). The medication treatment lasted for 6 weeks. Patients with sleep disorders could use benzodiazepines in combination, but benzodiazepines should not be taken continuously for more than 2 weeks, and other psychotropic medicines were not used in combination. The medicines used in this study were commonly used in the clinic, which had good safety and fewer side effects. The evaluations of patients’ side effects and adverse information by an outpatient doctor were collected regularly. If patients failed to comply with the agreement and stopped the medicines by themselves, they were deemed to have withdrawn from the study.

### Outcome Measures

The demographic data of the 3 groups were collected during the enrollment assessment. Obsessive-compulsive symptoms, the quality of life, and costs were collected 3 times: at baseline, during treatment (3 weeks), and after treatment (6 weeks). All assessments at baseline were offline, and subsequent assessments in the ICBT and TAU groups were conducted online. In addition, the study collected process indexes and recorded adverse events to assess feasibility and safety. The process indexes included the attendance rate, dropout rate, homework completion, and subjective satisfaction of patients.

#### Primary Outcome

Obsessive-compulsive symptoms were measured using a masked assessor–rated YBOCS [29] and the self-rating Florida Obsessive-Compulsive Inventory (FOCI) [33] as the primary outcome. The YBOCS is compiled by Goodman in the United States and contains 10 items to assess the severity of obsessive thoughts and compulsive behaviors. The scoring method adopts a 5-point scale of 0-4 points, and the total score range is 0-40 points, which has good reliability and validity. FOCI, a self-rating scale that contains 20 items, is used to assess the severity of obsessive-compulsive symptoms within 1 month. The first 15 items evaluate symptoms with yes/no questions, and the last 5 items evaluate the severity of symptoms on a 5-point scale of 0-4 points. After treatment, a YBOCS score reduction rate of 35% or more was considered effective treatment and a YBOCS score of less than 8 was classified as remission.

#### Secondary Outcome

The quality of life was measured using the EQ-VAS score of the EQ-5D [34] as the secondary outcome. The EQ-5D is a widely used scale to survey the living conditions of subjects. VAS is a 20-cm-long vertical visual scale, with a score of 100 at the top representing “the best health condition in one’s own mind” and a score of 0 at the bottom representing “the worst health condition in one’s own mind.” The degree of change in the quality of life of the 3 groups after treatment was assessed.

#### Cost Data

The study designed the Sinicized Cost Questionnaire based on Trimbos and the Institute of Medical Technology Assessment Cost Questionnaire for Psychiatry [35], which is a self-rated questionnaire about cost-effectiveness analyses, including both direct and indirect costs. Patients were asked to fill out the questionnaire themselves based on the bill, under the guidance of trained evaluators. The Sinicized Cost Questionnaire assesses the cost within a month.

Direct costs included registration, psychological treatment, medication, and rehabilitation. Indirect costs included transportation expenses, accommodation expenses, food expenses, medical and health care expenses due to illness, and the loss of productivity for patients and their families. To avoid confusion regarding the inability to work/study due to illness with unemployment/dropout, only the time spent in the hospital for consultation and treatment was counted as the number of days the patient was unable to work/study.

The cost-effectiveness analysis (CEA) method was used to measure the spending of the 3 groups. The results of the total cost divided by the reduction in the YBOCS total score reflected the cost required to obtain each unit of curative effect.

### Sample Size

The statistical method suitable for this study was 2-way (1 within-subject and 1 between-subject factor) repeated-measures ANOVA. According to the theoretical considerations [36] and the results of pilot data [24], the significance level was set as α=.05, the statistical test power was 1 – β = 80%, while effect size d=0.8. We used the formula for calculating the sample size of a noninferiority trial:



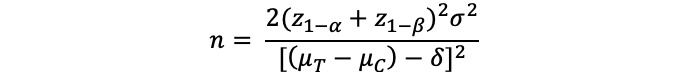



and the minimum sample size was nl=n2=n3=23. Considering the dropout rate and clinical operability, the TAU, ICBT, and CBGT groups should each have had 33 patients, so finally, 99 patients meeting the criteria were included.

### Randomization and Blinding

Random sequences were generated in Microsoft Excel with the RAND function, and all eligible patients were randomized 1:1:1 to the ICBT, CBGT, and TAU groups. The evaluators were blind to the patients’ treatment. At the same time, patients were asked not to mention the psychotherapeutic conditions to the evaluator during assessment. The arrangement of the time and location of the assessments for the 99 patients was the responsibility of the coordinator, who did not disclose the grouping of patients.

### Statistical Analysis

Statistical data were analyzed with SPSS 25.0 (IBM Corporation). All outcome analyses were conducted according to the intention-to-treat principle, and the missing data were dealt with using conditional mean imputation [37]. Repeated-measures ANOVA was used for continuous data to compare efficacy differences within/between groups. The data passed the tests of normality, the Mauchly test of sphericity, and epsilon correction [38], meeting the prerequisite of repeated-measures ANOVA. To calculate effect sizes, mean differences (95% CI) were used. One-way ANOVA was used to compare the differences in the attendance rate, homework completion, subjective satisfaction, and costs between groups, and specific differences in costs between groups were analyzed using the least significant difference (LSD) as the post hoc test. The chi-square test was performed to test for differences in the dropout rate, effective treatment rate, and clinical cure rate between groups. *P*<.05 was considered statistically significant.

The study repeated the analysis for the primary outcome using 2 different approaches as sensitivity analyses to assess the robustness of our conclusions. The first approach was complete case analysis. In this situation, only cases that completed all the follow-up measures (n=80, 86.0%) were analyzed, while cases with missing values were removed. The second approach was analysis with the addition of covariates. In this case, demographic variables, such as age, years of education, age at first onset, and total duration of the disease, were included as covariates in the repeated-measures ANOVA.

In cost analyses, the total cost was the sum of direct and indirect costs. The total direct/indirect cost of each group was obtained by adding the direct/indirect cost provided by each group. To quantify the cost of lost productivity in the indirect cost, the study averaged China's 2018 annual per capita income (renminbi [RMB] 27,996 [US $4235.40]) for 365 days (RMB 76.70 [US $11.60]) and then multiplied it by the number of patient working days lost. The mean (95% CI) and SD of costs were described. The cost-effectiveness ratio (CER) for each group was the result of the total cost divided by the total reduction in the YBOCS score, which was the amount of money spent for each reduction in the YBOCS score. The incremental cost-effectiveness ratio (ICER) took the cost of the group with the least expenditure as the standard to compare the additional expenditure of the other groups per reduction in the YBOCS score. The yuan (RMB) is the national currency of the People’s Republic of China, and the average exchange rate was US $1.00=RMB 6.61 for 2018.

## Results

### General Comparison

**Figure 1:**
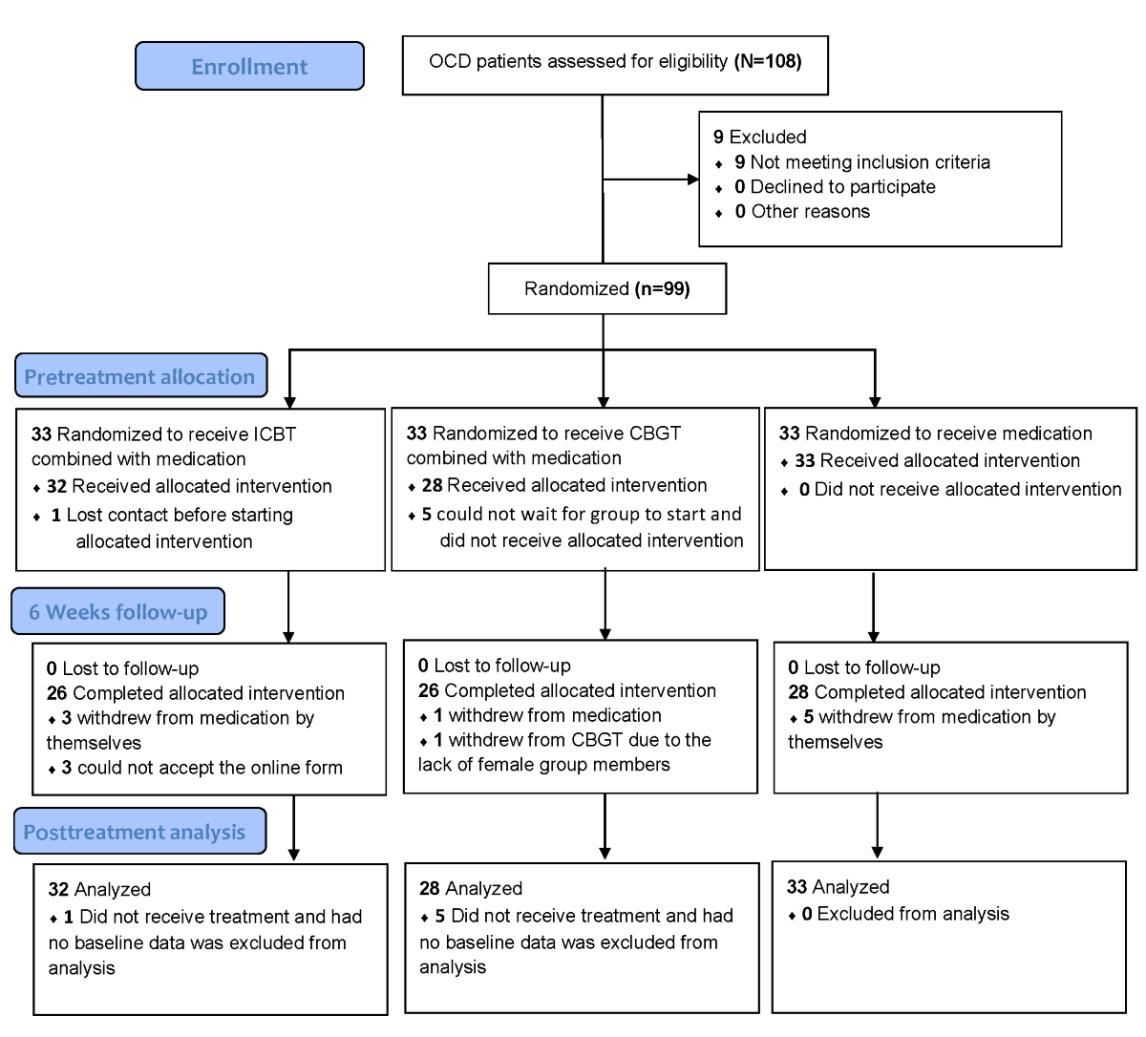
Participant flow in a study of the effect of ICBT vs CBGT vs TAU on OCD symptoms in adults. CBGT: cognitive behavioral group therapy; ICBT: internet-based cognitive behavioral therapy; TAU: treatment as usual; OCD: obsessive-compulsive disorder.

A total of 93 subjects (n=56, 60.2%, men; n=37, 39.8%, women) were included in the analysis. Participant flow and reasons for dropout throughout the trial are shown in Figure 1. The demographic data of the 3 groups at baseline are shown in Table 2. There were no significant differences in age (*P*=.34), gender (*P*=.37), years of education (*P*=.50), and total duration of the disease (*P*=.21) among the 3 groups.

**Table 2:** Demographic data of the 3 groups at baseline (N=93).

Characteristics		CBGT^a^ group (n=28)	ICBT^b^ group (n=32)	TAU^c^ group (n=33)
**Gender, n (%)**				
	Male	15 (53.6)	18 (56.3)	23 (69.7)
	Female	13 (46.4)	14 (43.7)	10 (30.3)
**Marital status, n (%)**				
	Unmarried	14 (50.0)	20 (62.5)	22 (66.7)
	Married	14 (50.0)	12 (37.5)	11 (33.0)
**Age (years)**				
	Mean (SD)	30.29 (8.09)	29.16 (6.35)	27.52 (7.73)
	Min-max (years)	18-48	19-45	18-45
Years of education, mean (SD)		13.82 (2.79)	13.81 (3.31)	14.55 (2.41)
**History of previous treatment, n (%)**				
	Psychiatric medications	28 (100)	32 (100)	33 (100)
	Traditional Chinese medicine	0	0	1 (3.0)
	Psychological treatment	0	0	1 (3.0)
Duration of SSRI^d^ treatment>8 weeks, n (%)		26 (92.9)	24 (75.0)	29 (87.9)
**Present onset form, n (%)**				
	Acute (within a month)	1 (3.6)	2 (6.3)	6 (18.2)
	Subacute	1 (3.6)	5 (15.6)	5 (15.2)
	Chronic (>3 months)	26 (92.9)	25 (78.1)	22 (66.7)
Total duration of the disease, mean (SD)		7.71 (6.71)	6.06 (6.18)	8.96 (6.65)
Age at first onset, mean (SD)		22.54 (6.61)	23.78 (7.25)	18.85 (6.53)
**Disease progression, n (%)**				
	Persistent	23 (82.1)	28 (87.5)	16 (48.5)
	Intermittent	5 (17.9)	4 (12.5)	17 (51.5)
Family history, n (%)		3 (10.7)	1 (3.1)	8 (24)

^a^CBGT: cognitive behavior group therapy.

^b^ICBT: internet-based cognitive behavior therapy.

^c^TAU: treatment as usual (conventional medical treatment).

^d^SSRI: selective serotonin reuptake inhibitor.

### Feasibility and Safety

In the ICBT group, 7 (21.2%) of 33 patients dropped out (n=3, 42.9%, men): 1 (14.3%) patient lost contact before the start of treatment, 3 (42.9%) patients discontinued the medication because their condition improved, and 3 (42.9%) patients withdrew because they could not receive online treatment. In the CBGT group, 7 (21.2%) of 33 patients dropped out (n=4, 57.1%, men): 5 (71.4%) patients dropped out because they could not wait until the treatment group was assembled, 1 (14.3%) patient withdrew because they discontinued medication themselves, and 1 (14.3%) female patient dropped out due to the lack of female patients in the group. In the TAU group, 5 (15.2%) patients dropped out (n=3, 60.0%, men) because they discontinued medication themselves. There was no statistically significant difference in the dropout rate among the 3 groups (*χ*^2^_2_=0.52, *P*=.77).

In conclusion, a total of 19 (19.2%) of 99 patients dropped out from the 3 groups during treatment. Of them, 6 (31.6%) patients did not participate in the treatment (n=5, 83.3%, in the CBGT group; n=1, 16.7%, in the ICBT group) and had no baseline data. The remaining 13 (68.4%) patients dropped out in the middle of treatment (n=2, 15.4%, in the CBGT group; n=6, 46.2%, in the ICBT group; and n=5, 38.4%, in the TAU group). According to the intention-to-treat principle, we replaced the of these 13 (68.4%) patients with the mean imputation, and the final effective data of 93 patients (ICBT: n=32, 34.4%; CBGT: n=28, 30.1%; TAU: n=33, 35.5%) were considered.

The results of the 7-level satisfaction scale evaluation of the 3 groups were as follows:

- ICBT group: 3-week mean 5.73 (SD 0.96); 6-week mean 5.77 (SD 0.82)
- CBGT group: 3-week mean 5.19 (SD 0.94); 6-week mean 5.30 (SD 0.97)
- TAU group: 3-week mean 4.86 (SD 0.76); 6-week mean 4.67 (SD 0.81)

During the treatment (3 weeks), the satisfaction degree of the TAU (*P*=.001) and CBGT (*P*=.03) groups was significantly lower than that of the ICBT group (between 3 groups: *F*_2,90_=6.62, *P*=.002); after treatment (6 weeks), the satisfaction degree of the TAU group was significantly lower than that of the ICBT (*P*=.001) and CBGT (*P*=.01) groups (between 3 groups: *F*_2,90_=6.62, *P*=.002).

Among the 12 treatments, there was no significant difference between the ICBT (18.59%) and CBGT (13.14%) groups in the absence rate (*P*=.44). In addition, there was no significant difference between the ICBT (16.03%) and CBGT (9.62%) groups in the homework incomplete rate (*P*=.65).

This study recorded 2 adverse events: 1 (3.1%) patient in the ICBT group expressed anxiety and vertigo symptoms during the reading text of module training and finally terminated the treatment and withdrew from the study, while 1 (3.6%) female patient in the CBGT group felt uncomfortable and withdrew from the study due to the lack of female members in the group. No serious adverse events occurred in this study.

### Primary Outcome

At baseline, there were no statistically significant differences in the YBOCS score (*F*_2,90_=0.73, *P*=.49) and the FOCI score (*F*_2,90_=0.23, *P*=.80) between the 3 groups. The mean and SD of the YOBCS and FOCI scores as well as the results of repeated-measures ANOVA and effect sizes for the primary efficacy measure at every time point for the 3 groups are shown in Tables 3-4.

**Figure 2:**
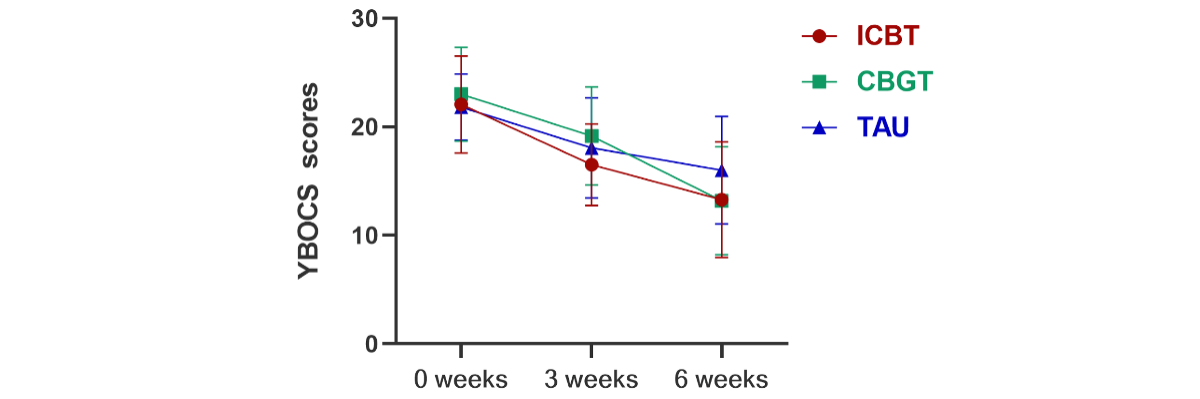
Primary outcome of YBOCS in a study of the effect of ICBT vs CBGT vs TAU on OCD symptoms in adults. CBGT: cognitive behavioral group therapy; ICBT: internet-based cognitive behavioral therapy; OCD: obsessive-compulsive disorder; TAU: treatment as usual; YBOCS: Yale-Brown obsessive-compulsive scale.

**Figure 3:**
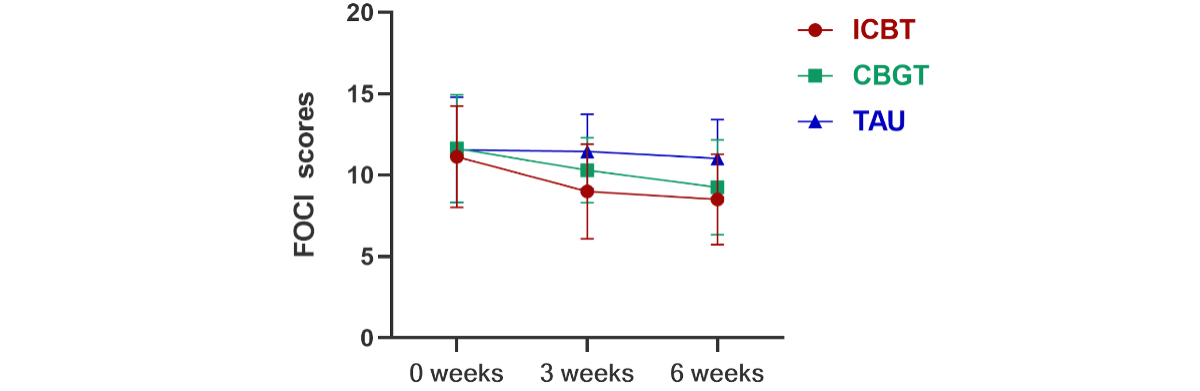
Primary outcome of FOCI in a study of the effect of ICBT vs CBGT vs TAU on OCD symptoms in adults. CBGT: cognitive behavioral group therapy; FOCI: Florida obsessive-compulsive inventory; ICBT: internet-based cognitive behavioral therapy; OCD: obsessive-compulsive disorder; TAU: treatment as usual.

There was a significant group×time interaction effect on the YBOCS score (*F*_4,84_=5.04, *P*=.001, η^2^=0.101) and the FOCI score (*F*_4,84_=3.33, *P*=.02, η^2^=0.069). Comparing the 3-week/6-week treatment with baseline, the YBOCS scores of the 3 groups significantly decreased (*P*<.001). The reduction in the YBOCS score of the ICBT and CBGT groups was more than that in the TAU group, but there was no significant difference between the groups. Comparing the 3-week/6-week treatment with baseline, the FOCI score of the ICBT and CBGT groups significantly decreased (*P*<.001), but there was no significant change in the TAU group. During treatment (3 weeks), the FOCI score of the ICBT group was significantly lower than that of the TAU group (*P*<.001). After treatment (6 weeks), the FOCI score of the ICBT (*P*=.001) and CBGT (*P*=.04) groups was significantly lower than that of the TAU group. Figures 2 and 3 show graphs of the estimated marginal means of YBOCS/FOCI scores at baseline, during treatment, and after treatment under 3 interventions. The treatment response rate of the 3 groups was different at 6 weeks (*P*=.001), and the specific treatment response and remission of the 3 groups at each time point are shown in Table 5.

**Table 3:** Mean and SD of the YOBCS^a^ and FOCI^b^ scores at baseline, during treatment (3 weeks), and after treatment (6 weeks) for the 3 groups.

Measure		Baseline, mean (SD)	During treatment, mean (SD)		After treatment, mean (SD)
**YBOCS**					
	ICBT^c^ group	22.06 (4.48)	16.50 (3.77)	13.28 (5.33)	
	CBGT^d^ group	23.00 (4.31)	19.14 (4.52)	13.18 (4.99)	
	TAU^e^ group	21.82 (3.06)	18.06 (4.61)	16.00 (4.96)	
**FOCI**					
	ICBT group	11.13 (3.11)	9.00 (2.92)	8.50 (2.77)	
	CBGT group	11.64 (3.31)	10.29 (2.00)	9.25 (2.91)	
	TAU group	11.55 (3.24)	11.45 (2.28)	11.03 (2.39)	

^a^YBOCS: Yale-Brown Obsessive-Compulsive Scale.

^b^FOCI: Florida Obsessive-Compulsive Inventory.

^c^ICBT: internet-based cognitive behavioral therapy.

^d^CBGT: cognitive behavioral group therapy.

^e^TAU: treatment as usual.

**Table 4:** Results of repeated-measures ANOVAs and effect sizes for the primary efficacy measure at baseline, during treatment (3 weeks), and after treatment (6 weeks) for the 3 groups.

Measure		Difference at 3 weeks, mean (95% CI), *P* value		Difference at 6 weeks, mean (95% CI), *P* value		*P* value
		Within group	Between group	Within group	Between group	Group×time interaction
**YBOCS^a^**						
	ICBT^b^ group	–5.56 (–7.33 to –3.79), <.001	N/A^c^	–8.78 (–10.95 to –6.61), <.001	N/A	.001
	ICBT group vs CBGT^d^ group	N/A	–2.64 (–5.36 to 0.08), .06	N/A	0.10 (–3.12 to 3.32), .99	N/A
	ICBT group vs TAU^e^ group	N/A	–1.56 (–4.17 to 1.05), .444	N/A	–2.72 (–5.80 to 0.37), .103	N/A
	CBGT group	–3.86 (–5.75 to –1.97), <.001	N/A	–9.82 (–12.14 to –7.50), <.001	N/A	N/A
	CBGT group vs TAU	N/A	1.08 (–1.62 to 3.78), .993	N/A	–2.82 (–6.02 to 0.37), .102	N/A
	TAU group	–3.76 (–5.50 to –2.02), <.001	N/A	–5.82 (–7.96 to –3.68), <.001	N/A	N/A
**FOCI^f^**						
	ICBT group	–2.13 (–3.39 to –0.86), <.001	N/A	–2.63 (–3.99 to –1.26), <.001	N/A	.02
	ICBT group vs CBGT group	N/A	–1.29 (–2.83 to 0.26), .135	N/A	–0.75 (–2.45 to 0.95), .851	N/A
	ICBT group vs TAU group	N/A	–2.46 (–3.93 to –0.98), <.001	N/A	–2.53 (–4.16 to –0.90), .001	N/A
	CBGT group	–1.36 (–2.71 to –0.01), <.001	N/A	–2.39 (–3.85 to –0.93), <.001	N/A	N/A
	CBGT group vs TAU group	N/A	–1.17 (–2.70 to 0.36), .198	N/A	–1.78 (–3.47 to –0.10), .04	N/A
	TAU group	–0.09 (–1.34 to 1.16), .99	N/A	–0.52 (–1.86 to 0.83), .99	N/A	N/A

^a^YBOCS: Yale-Brown Obsessive-Compulsive Scale.

^b^ICBT: internet-based cognitive behavioral therapy.

^c^N/A: not applicable.

^d^CBGT: cognitive behavioral group therapy

^e^TAU: treatment as usual.

^f^FOCI: Florida Obsessive-Compulsive Inventory.

**Table 5:** Treatment response and remission under 3 interventions after 6 weeks of treatment.

Time			ICBT^a^ group (n=32), n (%)		CBGT^b^ group (n=28), n (%)		TAU^c^ group (n=33), n (%)		*P* value^d^
**Treatment response rate^e^**									
	3 weeks	10 (31)		3 (11)		4 (12)		.06	
	6 weeks	20 (63)		22 (79)		11 (33)		.001	
**Remission rate^f^**									
	3 weeks	0		0		1 (3)		.40	
	6 weeks	4 (13)		5 (18)		1 (3)		.16	

^a^ICBT: internet-based cognitive behavioral therapy.

^b^CBGT: cognitive behavioral group therapy.

^c^TAU: treatment as usual.

^d^*P* values are the difference in the treatment response rate and the remission rate between the groups on the chi-square test under the 3 interventions.

^e^YBOCS score reduction rate≥35.0% after treatment was defined as the treatment response.

^f^After treatment, YBOCS score<8 was classified as remission.

### Secondary Outcome

**Figure 4:**
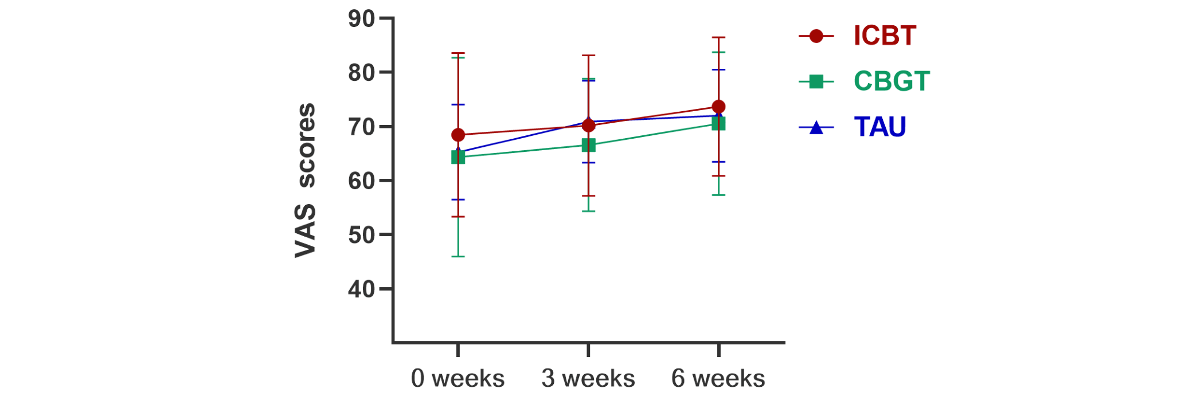
Secondary outcome of VAS in a study of the effect of ICBT vs CBGT vs TAU on OCD symptoms in adults. CBGT: cognitive behavioral group therapy; ICBT: internet-based cognitive behavioral therapy; OCD: obsessive-compulsive disorder; TAU: treatment as usual; VAS: visual analogue scale.

The VAS scores in the EQ-5D scale for the 3 groups had no significant differences at baseline (*F*_2,90_=0.62, *P*=.54), and the VAS scores of the 3 groups before and after treatment significantly improved (*P*<.001). The VAS scores of the ICBT group after treatment (mean 73.69, SD 12.78, 95% CI 69.07-78.30) improved compared with those at baseline (mean 68.47, SD 17.23, 95% CI 62.26-74.68). The VAS scores of the CBGT group after treatment (mean 70.54, SD 13.21, 95% CI 65.41-75.66) improved compared with those at baseline (mean 64.36, SD 18.41, 95% CI 57.22-71.49). The VAS scores of the TAU group after treatment (mean 72.00, SD 8.51, 95% CI 68.98-75.02) improved compared with those at baseline (mean 65.27, SD 8.81, 95% CI 62.15-68.40). In addition, the difference between groups was not significant after treatment (*F*_2,90_=0.56, *P*=.58). Figure 4 shows the estimated marginal means of VAS scores at baseline, during treatment, and after treatment under 3 interventions.

### Health Economics Analyses

#### Cost Analyses

After 6-week treatment, the total cost per person in the CBGT group was RMB 6678.45, 95% CI 4460.88-8896.01 [US $1010.36, 95% CI 678.87-1345.84]), that in the ICBT group was RMB 3308.81, 95% CI 2476.89-4140.73 [US $500.58, 95% CI 374.72-626.43]), and that in the TAU group was RMB 2259.61, 95% CI 2074.16-2445.05 [US $341.85, 95% CI 313.79-369.90]). There was a significant difference in the total cost between the 3 groups (*F*_2,90_=13.45, *P*<.001), and post hoc testing (LSD) showed that the total cost of the CBGT group was significantly higher than that of the ICBT (*P*<.001) and TAU (*P*<.001) groups. There were also significant between-group differences in the total direct (*F*_2,90_=29.80, *P*<.001) and total indirect (*F*_2,90_=3.57, *P*=.03) costs, and the direct cost of the CBGT group was significantly higher than that of the ICBT (*P*<.001) and TAU (*P*<.001) groups; the indirect cost of the CBGT group was significantly higher than that of the ICBT (*P*=.04) and TAU (*P*=.01) groups. See Table 6 for more cost data.

**Table 6:** Costs (RMB^a^/US $^b^) of the 3 groups after 6-week treatment.

Type of cost	CBGT^c^ group (n=28)			ICBT^d^ group (n=32)			TAU^e^ group (n=33)			Between-group comparison^f^	
	Total cost	Mean (95% CI)	Total cost		Mean (95% CI)	Total cost		Mean (95% CI)	*F*_2,90_ value		*P* value
Total cost	186,997.00/28,290.02	6678.45 (4460.88-8896.01)/1010.36 (678.87-1345.84)	105,882.00/16,018.46		3308.81 (2476.89-4140.73)/500.58 (374.72-626.43)	74,567.00/11,280.94		2259.61 (2074.16-2445.05)/341.85 (313.79-369.90)	13.45		<.001
Direct cost	124,844.00/18,887.14	4458.70 (3639.37-5278.03)/674.54 (550.59-798.49)	73,701.00/11,149.92		2303.16 (1717.54-2888.77)/348.44 (259.84-437.03)	50,246.00/7601.51		1522.61 (1382.19-1663.03)/230.35 (209.11-251.59)	29.80		<.001
Indirect cost	62,153.00/9,402.87	2219.75 (673.77-3765.83)/335.82 (101.93-569.72)	32,181.00/4868.53		1005.66 (635.49-1375.73)/152.14 (96.14-208.13)	24,321.00/3679.43		737.00 (632.77-841.06)/111.50 (95.73-127.24)	3.57		.03

^a^RMB: renminbi.

^b^The average exchange rate was US $1.00=RMB 6.61 for 2018.

^c^CBGT: cognitive behavioral group therapy.

^d^ICBT: internet-based cognitive behavioral therapy.

^e^TAU: treatment as usual.

^f^The *F* and *P* values are the results of between-group comparisons of costs using 1-way ANOVA among the 3 groups.

#### Cost-Effectiveness Analyses

After 6-week treatment, it cost RMB 376.80 (US $57.00) for the ICBT group to reduce the YBOCS score by 1, RMB 388.37 (US $58.75) for the TAU group, and RMB 679.99 (US $102.87) for the CBGT group. The ICBT group spent RMB 303.19 (US $45.97) less than the CBGT group and RMB 11.57 (US $1.75) less than the TAU group. See Table 7.

**Table 7:** Cost-effectiveness analysis (RMB^a^/US $^b^) of patients after 6-week treatment.

Group	Total cost (RMB/US $)	YBOCS^c^ average score		YBOCS total reduction score	CER^d^ (RMB/US $)	ICER^e^ (RMB/US $)
		Baseline	After treatment			
CBGT^f^	186,996.50/28,289.94	23.00	13.18	275.00	679.99/102.87	303.19/45.97
TAU^g^	74,567.00/11,280.94	21.82	16.00	192.00	388.37/58.75	11.57/1.75
ICBT^h^	105,882.00/16,018.46	22.06	13.28	281.00	376.80/57.00	0.00/0.00

^a^RMB: renminbi.

^b^The average exchange rate was US $1.00=RMB 6.61 for 2018.

^c^YBOCS: Yale-Brown Obsessive-Compulsive Scale.

^d^CER: cost-effectiveness ratio.

^e^ICER: incremental cost-effectiveness ratio.

^f^CBGT: cognitive behavioral group therapy.

^g^TAU: treatment as usual.

^h^ICBT: internet-based cognitive behavioral therapy.

### Therapist Time Consumption

This study did not include the time of therapists for cost calculation but collected the total time of the therapists occupied by the 3 groups of interventions. The results showed that patients in the ICBT group consumed a total of 1078 minutes of the therapist’s time during the 6-week treatment, with an average of 32.67 (SD 13.37) minutes per person; the CBGT group consumed a total of 2880 minutes of the therapist’s time during the 6-week treatment, with an average of 102.86 (SD 0) minutes per person. Compared to the CBGT group, the ICBT group saved a total of 1802 minutes of the therapist’s time, with an average of 70.19 (SD 13.37) minutes saved per person.

### Sensitivity Analysis

The study performed complete case analysis (n=80, 86.0%) and analysis with the addition of covariates, and the results of the 2 sensitivity analyses were consistent with those of the primary analyses, suggesting the robustness of our conclusions.

## Discussion

### Principal Findings

This study was an RCT of ICBT, CBGT, and conventional medical treatment for OCD in China, which aimed to evaluate the efficacy and cost-effectiveness of therapist-guided ICBT for Chinese patients with OCD. All 3 groups had curative effects and improved quality of life after treatment for 6 weeks, and the ICBT group was more cost-effective than the other groups. The satisfaction of patients was high, and there were no serious adverse events.

In terms of efficacy, the significant efficacy of SSRIs was consistent with the results shown in earlier studies [15]. Most patients had been taking the medication stably before the start of the experiment, which made the treatment become effective in the short term. ICBT/CBGT combined with medical treatment showed significant efficacy and had a higher mean difference than the medicine alone (although the difference between groups was not statistically significant), suggesting that ICBT and CBGT could effectively improve OCD symptoms with comparable efficacy, which was similar to previous studies [18] and our preexperiment [26]. The frequency of ICBT and CBGT in the study was twice a week, which might be the reason for the obvious effect of treatment after 6 weeks. Although the content and structure of treatment were standardized, the duration of treatment might be different (commonly 6, 8, 10, 12, and so on), and future research can explore whether the group intensity affects the efficacy of ICBT. In addition, the ICBT group showed a higher mean difference than the CBGT and TAU groups during treatment (3 weeks), which was consistent with the results of our preexperiment [26]. The possible reason is that the training time of the psychological education module in the ICBT group was relatively short. The patients in the ICBT group had already completed several ERP exercises in the third week, while the CBGT group just completed 1 ERP exercise. Earlier studies have shown that the number of ERP training sessions is positively correlated with the effect of treatment [39]. However, after treatment, the effect for the 2 groups was equivalent, indicating that although the 2 groups had different onset times, the interventions were overall effective in the entire 6 weeks of treatment. Therefore, we can synchronize the ERP training process of ICBT and CBGT in future research and studies so as to better compare the efficacy of the 2 treatment methods at various time points, which will also provide more possibilities for the subsequent combination or replacement of the 2 treatment methods.

A difference between the results of the YBOCS and FOCI was found in this study. After treatment (6 weeks), the FOCI score of the ICBT and CBGT groups was significantly lower than that of the TAU group, while this difference was not detected in the YBOCS. It was probably because the YBOCS is an assessor-rated scale, while FOCI is a self-rating scale. Patients may have more acute observations of the changes in their own obsessive-compulsive symptoms and experience. This might be more sensitively reflected in the FOCI score after ICBT/CBGT rather than the blind assessor–rated YBOCS. In addition, the satisfaction degree of treatment in the ICBT/CBGT group was higher than that in the TAU group, which might also affect the self-rating scores.

With regard to feasibility, the results showed that the TAU group had the lowest dropout rate, and the ICBT group had the same dropout rate as the CBGT group. Half of the patients dropped out in the ICBT group due to discomfort with reading text on an electronic screen. Completing therapy and homework by reading and typing on the keyboard might be unfriendly to some people, especially older patients. A patient in the ICBT group withdrew from the study because of anxiety and vertigo symptoms during training and reading. It was difficult for ICBT when the patient was not accustomed to using the computer, lacked motivation for treatment, or had too severe symptoms. Patient compliance did correlate with treatment modality, and some patients who dropped out reported that they were not used to online therapy. A previous paper indicated that a single human-computer interaction might lack face-to-face treatment, resulting in decreased compliance of patients during treatment [40]. It may be helpful to enhance the interactivity of the ICBT course. Therefore, although ICBT has the advantages of being easy to operate, avoiding stigma, and being free from geographical restrictions, ICBT cannot be applied to all patients. However, the satisfaction of the ICBT group was still higher than that of the other 2 groups, indicating that the form of intervention could be acceptable to most patients, which is consistent with previous studies [7]. Similarly, CBGT also has formal limitations. The patients who dropped out in the CBGT group exited while waiting for the establishment of the group, because they had to wait for about 3 weeks due to insufficient members. In addition, a female patient in the CBGT group felt uncomfortable due to the lack of female members in the group and eventually terminated her treatment. In addition, some patients would be absent from treatment due to time or transportation conflicts. In general, ICBT is more suitable for patients who are highly motivated and accustomed to online operations, while CBGT is more suitable for patients who have much spare time and transportation and are willing to grow in groups. To avoid the respective limitations of the 2 therapies as much as possible, therapists can provide appropriate treatment methods according to the characteristics of patients and try to combine the 2 treatments in clinical practice to provide a more personalized plan.

Health economics results showed that ICBT is significantly less expensive than CBGT and was the most cost-effective option of the 3. The cost savings of the ICBT group might be achieved by less transportation expenses, accommodation expenses, food expenses, and productivity loss because patients in the ICBT group could choose their own ERP practice time at home without extra transportation, accommodation, and work absence compared to the CBGT group. From health organizational perspectives (ie, therapist time) due to the online technology and the therapist-guided self-help mode, compared to the CBGT group, the ICBT group saved time for therapists, with an average of 76.43 minutes per person, which may help solve the human resource shortage in mental health services in China. Nowadays, the burden on mental health in various countries is increasing. For example, the United States spent more than US $300 billion on mental health each year [41], Sweden spent US $10.5 billion on mental health diseases each year [42], and many countries still have problems with patients not receiving effective treatment due to high social costs [39]. In addition, a multicenter survey based on hospitals showed the annual cost of OCD in China was estimated to be US $5.34 billion [43]. In this case, as an economical and effective method, therapist-guided ICBT may become an important treatment approach in the mental health field, especially in the field of OCD. Although ICBT has been shown to have a higher absence rate and dropout rate in studies, patient compliance can be improved through psychoeducation, regular contact of therapists with patients, and a combination of online and offline therapy, which can increase the flexibility of therapy, while keeping costs low.

### Limitations

This study has a few limitations. First, the dose and duration of patients’ SSRI treatment before enrollment were not clearly recorded, which were variables that could be included in the analysis. In addition, 8 weeks should be used as a standard for stable medication to screen subjects to reduce the impact of medication duration on the trial. Furthermore, the mean imputation may underestimate the variability of the data, although the sensitivity analysis of complete cases (n=80) showed consistent results with the primary analyses. In terms of health economics, it would be more comprehensive to include the cost of treatment space and therapists’ time in the cost analyses in the future. For example, CBGT requires renting treatment rooms and takes more time of therapists, which may lead to more costs for CBGT.

Based on the results of the equivalent efficacy of ICBT and CBGT, researchers and physicians can provide ICBT as a treatment option for patients in clinical practice, try to enhance the interactivity of ICBT courses or develop a combination of online and offline treatment methods, and gradually carry out some in-depth research in the future. For example, researchers should develop and validate the feasibility and efficacy of combined online and offline treatments. In addition, researchers can try to explore ICBT based on individualized assessment and make better use of the advantages of the online form through a step care model. Multicenter studies need to be carried out in the future. In addition, some studies have focused on the long-term efficacy of ICBT [21,22], which requires repeated verification.

Second, this study only focused on therapist-guided ICBT and did not explore self-guided ICBT. The latest research has verified the feasibility of self-guided ICBT (unguided ICBT) [44,45], but because of the high degree of freedom and lack of standardization of this method, it is still necessary to continue research.

### Conclusion

Therapist-guided ICBT combined with medication is as effective as face-to-face CBGT combined with medication for OCD. ICBT combined with medication is more cost-effective than CBGT combined with medication and conventional medical treatment. It is expected to become an efficacious and economic alternative for adults with OCD when face-to-face CBGT is not available.

## Appendix

Multimedia Appendix 1 CONSORT-eHEALTH checklist (V 1.6.1).

## Abbreviations

CBGT: cognitive behavioral group therapy
CBT: cognitive behavioral therapy
CBTC: Cognitive Behavioral Therapy China
CER: cost-effectiveness ratio
EQ-5D: EuroQol 5D Questionnaire
EQ-VAS: EuroQol Visual Analogue Scale
ERP: exposure and response prevention
ffCBT: face-to-face CBT
FOCI: Florida Obsessive-Compulsive Inventory
ICBT: internet-based cognitive behavioral therapy
ICER: incremental cost-effectiveness ratio
LSD: least significant difference
OCD: obsessive-compulsive disorder
RMB: renminbi
RCT: randomized controlled trial
SSRI: selective serotonin reuptake inhibitor
TAU: treatment as usual
VAS: Visual Analogue Scale
YBOCS: Yale-Brown Obsessive-Compulsive Scale
